# Cell Wall Modifications in Giant Cells Induced by the Plant Parasitic Nematode *Meloidogyne incognita* in Wild-Type (Col-0) and the *fra2* *Arabidopsis thaliana* Katanin Mutant

**DOI:** 10.3390/ijms20215465

**Published:** 2019-11-02

**Authors:** Christianna Meidani, Nikoletta G. Ntalli, Eleni Giannoutsou, Ioannis-Dimosthenis S. Adamakis

**Affiliations:** 1 Department of Botany, Faculty of Biology, National and Kapodistrian University of Athens, 157 84 Athens, Greece; sbi1200150@gmail.com (C.M.); egianno@biol.uoa.gr (E.G.); 2 Department of Pesticides Control and Phytopharmacy, Benaki Phytopathological Institute, 14561 Athens, Greece; nntali@agro.auth.gr

**Keywords:** arabinan, callose, cell walls, giant-cells, homogalacturonan, katanin, root-knot nematodes

## Abstract

*Meloidogyne incognita* is a root knot nematode (RKN) species which is among the most notoriously unmanageable crop pests with a wide host range. It inhabits plants and induces unique feeding site structures within host roots, known as giant cells (GCs). The cell walls of the GCs undergo the process of both thickening and loosening to allow expansion and finally support nutrient uptake by the nematode. In this study, a comparative in situ analysis of cell wall polysaccharides in the GCs of wild-type Col-0 and the microtubule-defective *fra2* katanin mutant, both infected with *M. incognita* has been carried out. The *fra2* mutant had an increased infection rate. Moreover, *fra2* roots exhibited a differential pectin and hemicellulose distribution when compared to Col-0 probably mirroring the *fra2* root developmental defects. Features of *fra2* GC walls include the presence of high-esterified pectic homogalacturonan and pectic arabinan, possibly to compensate for the reduced levels of callose, which was omnipresent in GCs of Col-0. Katanin severing of microtubules seems important in plant defense against *M. incognita*, with the nematode, however, to be nonchalant about this “katanin deficiency” and eventually induce the necessary GC cell wall modifications to establish a feeding site.

## 1. Introduction

Root-knot nematodes (RKN; *Meloidogyne* spp.) are obligatory parasites that inhabit plant roots, nursing from specially modified host cells to complete their life cycle [1]. Of major interest is the *Meloidogyne incognita* species that infests crops of high economic interest, causing worldwide agricultural yield production reduction [2,3,4]. *M. incognita* stage 2 juveniles (J2) enter the host plant close to the root tip; they migrate towards the elongation zone anchor to the root central cylinder, become sedentary and after bypassing host defenses, establish a feeding site [5]. Nematode signals direct root cylinder parenchyma cells to differentiate into multinucleate and metabolically active giant cells (GCs) that resemble transfer cells [1], allowing *M*. *incognita* to withdraw nutrients from the plants’ conducting tissues. During root invasion, *M. incognita* juveniles express numerous genes encoding cell wall-degrading enzymes and virulence effectors to enter, migrate into roots, establish feeding site and eventually induce GC formation [6,7]. At the end, the feeding site consists of several GCs enclosed in a macroscopically visible gall, a typical characteristic of RKN infestation. The second stage juveniles after entering the root, hatch to pass through several developmental stages (J3, J4) and finally reach to a stage of a female able to lay many eggs inside an egg sack [8]. The male leaves the root and does not harm host plants [9].

During *M*. *incognita* invasion the plant defense mechanisms are activated [10]. The plant cell wall plays a fundamental role in this process [11,12]. Plant cell walls differentiate from the primary walls of growing cells to the secondary walls, deposited after the end of cell expansion, which gradually thicken. Primary and secondary cell walls basically consist of cellulose, matrix polysaccharides and structural proteins, while in some cases secondary cell walls are lignified [13]. Matrix polysaccharides which coexist with cellulose microfibrils, are combinations of xyloglucans, hetero- xylans, heteromannans and the pectin groups of homogalacturonans (HGs) and rhamno- galacturonans [14]. Moreover, glycoproteins such as extensins and arabinogalactan-proteins (AGPs), take part in the structure and signaling properties of plant cell wall [15].

The cell walls of GCs induced by RKN undergo the process of both thickening and loosening to allow expansion and finally support nutrient uptake by the nematode [1,16]. In another plant parasitic nematode, namely the cyst nematode of the genus *Heterodera* spp., syncytia cells were induced, with profuse highly methyl-esterified HGs (MPHGs), xyloglucans and arabinans, to furnish the necessary flexibility to the structure for growth and preservation of turgor pressure of the syncytia [17].The highly-MPHGs, xyloglucans and arabinans are important components of *M*. *incognita* nematode-induced feeding giant cell cell walls, as well and also facilitate nourishment absorption [11,17]. The abundance of HGs may also be related to an increased requirement for flexibility of the giant cell walls [11]. As it has also been observed in guard cell walls, the increased presence of HGs in their cell walls helps to maintain their flexibility during changes in cell volume and shape [18].

Judging from the above, one can easily assume that cell wall defects could influence the RKN infection. Efforts towards this notion have already being made and a wide range of *Arabidopsis thaliana* mutants compromised in specific cell wall components were used [11]. Pectic HG-, manann-, arabinan-, arabinogalactan- and β-galactosidase-related mutants in particular were used to explore the impact of these mutations on *M*. *incognita* infection [11]. Along with the pre-mentioned proteins and matrix polysaccharides, cellulose microfibrils are critical for all aspects of plant morphogenesis [19], and would be interesting to investigate whether any deficiency in cellulose could affect RKN infection rates. In this context, katanin mutations (*fra2, lue1, bot1, erh3, ktn1)* cause a dramatic reduction in cell length and an increase in cell width [20]. This altered cellular morphology is a result of altered cellulose microfibril deposition caused by aberrant microtubule (MT) patterning, finally leading to a reduced amount of cellulose [21]. 

In particular, the *fra2* katanin mutant, has a point mutation (in the seventh exon, the A at nucleotide residue 2329 was deleted, resulting in a frameshift of the coding sequence, which caused the appearance of a premature stop codon) at the *AtKSS* gene which encodes the p60 subunit of katanin, a MT severing protein [22]. The mutant displays a semi-dwarf phenotype, and all its organs are shorter than those of the wild type. These morphological deficiencies of *fra2*, especially in axial organs, were attributed to the failure of cortical MTs to achieve a uniform transverse orientation in elongating cells [22]. This, in turn, results to a rather isotropic cell growth, responsible for the characteristic phenotype of the above mutant [20,23]. Moreover, in *fra2* it was shown that the amount of cellulose and hemicelluloses are reduced, and the concentration of lignin is increased in total cell wall extracts of the stems [22]. The reduced synthesis of cell wall components apparently occurs in both primary and secondary walls, as revealed by transmission electron microscopy [21]. So *fra2* are stubby-looking plants which display fragility of all of their organs and particularly of the stems. [20,22,23].

According to the above, the objective of this work was to compare in situ for the first time the cell wall polysaccharides in the giant cells of both wild-type (Col-0) and the *fra2* katanin mutant, both infected with *M. incognita.* An effort was made in order to understand in detail the structure of the cell walls of giant cells formed 21 days post-infection (21 dpi) with *M*. *incognita*. Also the role of the altered MT network and the fragility of the *fra2* mutant was investigated. A set of monoclonal antibodies was used to locate the major wall components in giant cells induced by RKN. The role of *fra2* mutation in GCs cell wall modifications and in nematode infection is discussed.

## 2. Results

### 2.1. Cell Wall Hemicellulose and Pectin Epitope Analysis of Uninfected Roots

**Figure 1:**
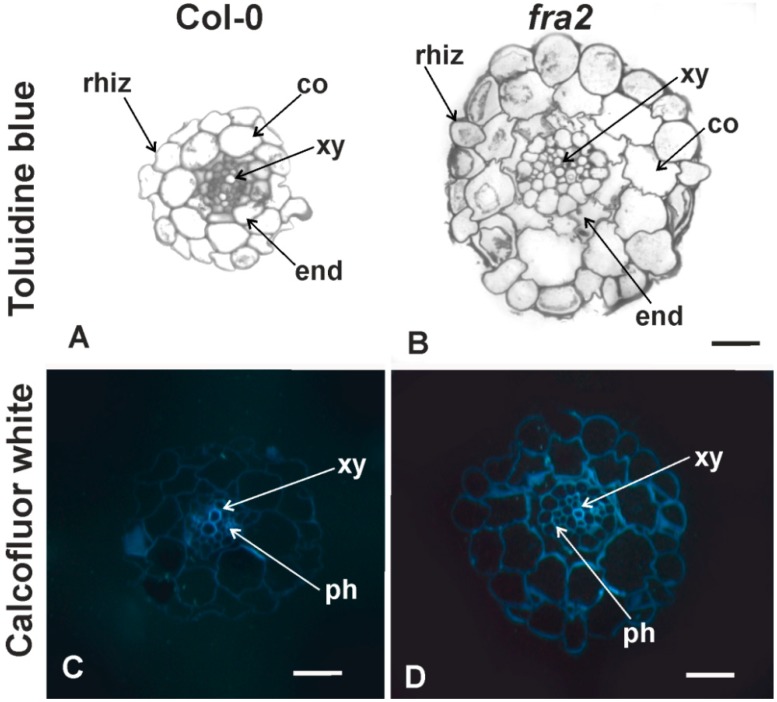
Comparative anatomy of Col-0 (**A**,**C**) and *fra2* (**B**,**D**) uninfected primary root transverse sections. Transverse sections either toluidine blue (**A**,**B**) or calcofluor-white stained (**C**,**D**) are depicted. Rhiz, rhizodermis; co, cortical cells; phy, phloem; xy, xylem elements; end, endodermis. Scale bar: 40 µm (**A**,**B**), 20 μm (**C**,**D**).

Toluidine blue staining further confirmed the already observed [24] differences of *fra2* and wild type root anatomical features. Generally, the primary *A. thaliana* root anatomy is similar to *fra2* and Col-0 roots (Figure 1) as it has been already reported [22]. Cell division zone of *fra2* roots is shorter than that of the wild type. Furthermore, due to an increase in cell width, an overall increase in root diameter could be observed (Figure 1B cf. Figure 1A). The mean diameter of Col-0 roots was 81.5 ± 0.9 μm, while mean root diameter of *fra2* plants was 146.3 ± 0.5 μm (± standard error; *n* = 10). All epitopes analyzed were present in the uninfected roots. Cellulose staining (calcofluor-white staining) was more intense in *fra2* (Figure 1D cf. Figure 1C).

**Figure 2:**
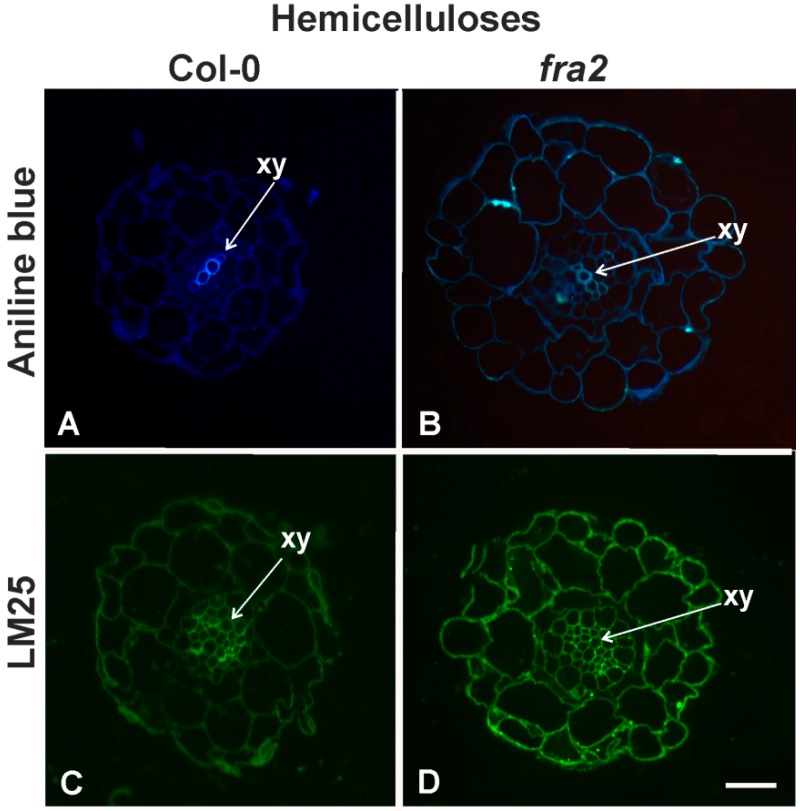
Hemicellulose distribution: Callose (**A**,**B**) staining and xyloglucans (**C**,**D**) immunolabelling in Col-0 (**A**,**C**) and *fra2* (**B**,**D**) uninfected primary root transverse sections. Aniline blue staining is stronger on the *fra2* root section (**B**) when compared to the Col-0 section, where it was restricted to the xylem elements (**A**). The LM25 antibody was used to localize xyloglucan. In the Col-0 root its signal was restricted to the vascular cylinder (**C**); while in *fra2* root section it had a broader distribution, occupying all the root tissues (**C** cf. **D**). Xy, xylem elements. Scale bar: 40 µm (**A**–**D**).

However, some epitopes displayed different distribution patterns between Col-0 and *fra2* plants. In particular, callose staining (aniline blue) was more intense in *fra2* (Figure 2B), while in wild-type plants callose signal was strong in the xylem elements (Figure 2A). The xyloglucans epitope recognized by LM25 had a more expanded cortex cell distribution in *fra2*, in contrast to the vascular cylinder cell distribution pattern of it in the Col-0 plants (Figure 2D cf. Figure 2C). 

**Figure 3:**
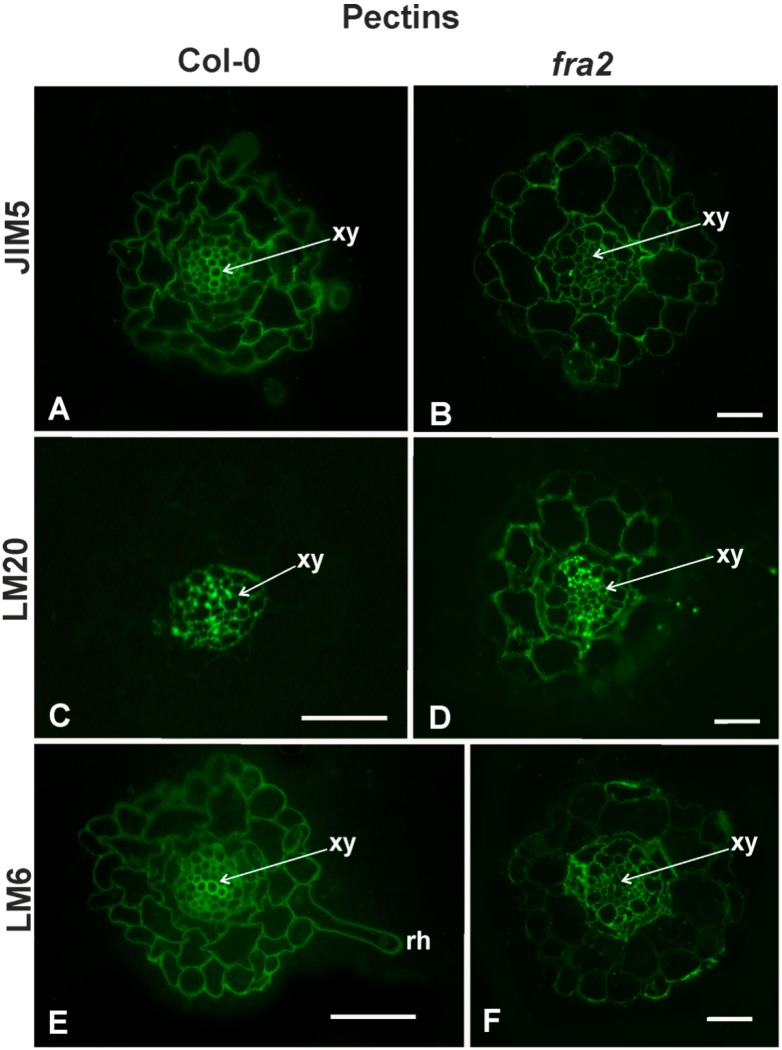
Pectin immunolabelling in Col-0 (**A**,**C**,**E**) and *fra2* (**B**,**D**,**F**) uninfected primary root transverse sections. DeSPHG localized by the JIM5 antibody did not seem to differ between Col-0 and *fra2* roots (**B** cf. **A**). Methyl-esterified MPHGs localized by the LM20 antibody had a boarder distribution in the *fra2* roots compared to Col-0 (**D** cf. **C**). Arabinan localized epitopes (LM6) were restricted, to the vascular cylinder, signal in *fra2* roots (E) but more broadly distributed in Col-0 roots (**F**). Xy, xylem elements; rh, roothair. Scale bars: (**A**,**B**) 40 µm, (**C**,**E**) 40  µm, (**D**,**F**)  40 µm.

Pectic polysaccharide and arabinogalactan-protein epitopes also had a different distribution in uninfected Col-0 and *fra2* root sections. The demethylesterified HG (DeSPHG) epitope recognized by JIM5 did not seem to differ between Col-0 and *fra2* (Figure 3A cf. Figure 3B). Highly MHGs recognized by LM20 were present only in the vascular cylinder of the Col-0 while in the *fra2* root sections their distribution expanded also to the cortex cells (Figure 3C cf. Figure 3D). Arabinan epitopes (LM6) had a more expanded distribution in wild-type roots, found in all of the root tissues, while in *fra2* their distribution was mostly restricted to the vascular cylinder (Figure 3E cf. Figure 3F).

### 2.2. Hemicellulose and Pectin Distribution Patterns of Giant Cell Walls of Col-0 and fra2 Roots

**Figure 4:**
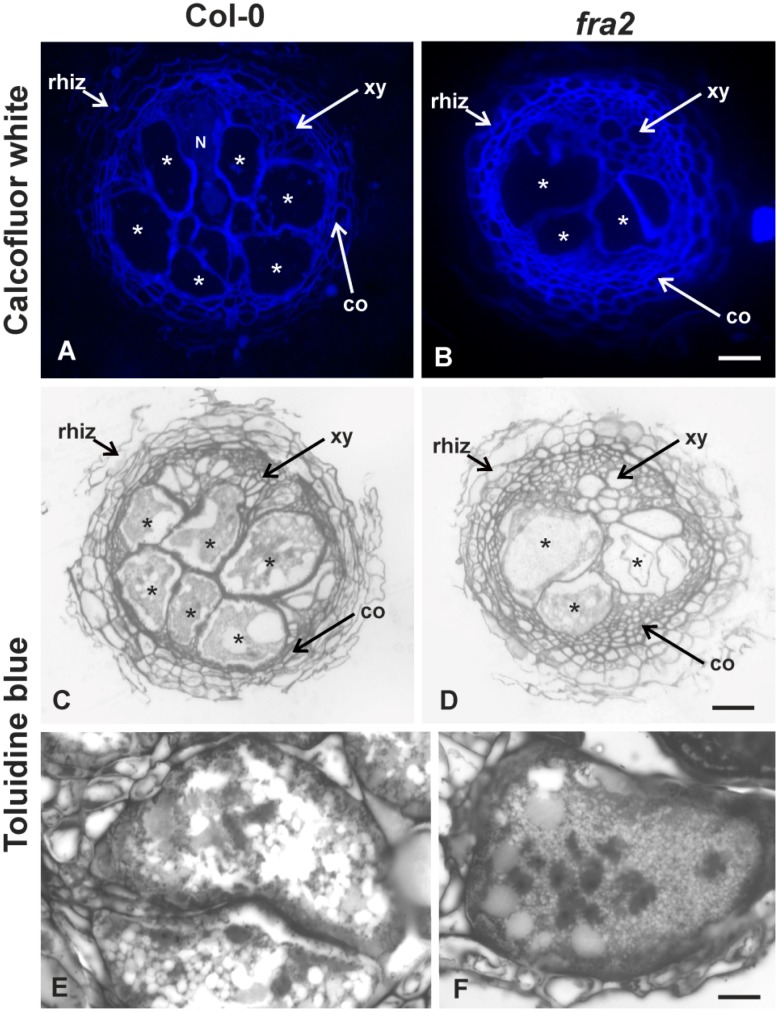
Comparative anatomy of root-knots of Col-0 (**A**,**C**) and *fra2* (**B**,**D**) 21 dpi by *M*. *incognita*, and nematode-induced giant cells (**E**,**F**). Transverse sections either calcofluor-white- (**A**,**B**) or toluidine blue-stained (**C**,**D**) are depicted. Asterisks indicate nematode-induced giant cells; N, indicates female *M. incognita*; co, cortical cells; xy, xylem vessels; rhiz, rhizodermis. Scale bars: (**A**,**B**) 40 µm, (**C**,**D**) 40 μm, (**E**,**F**) 40 μm.

**Figure 5:**
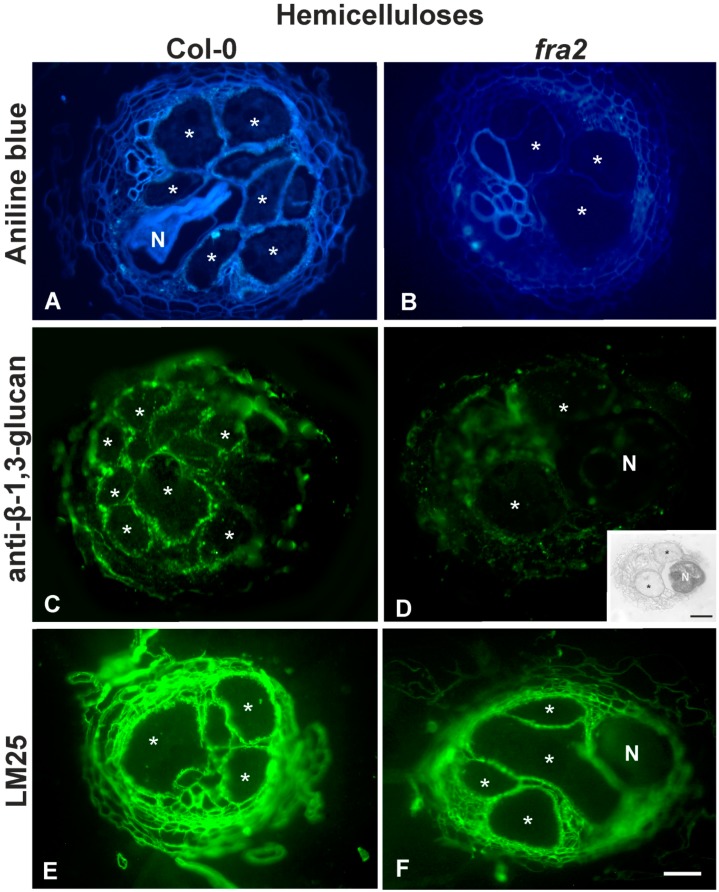
Hemicellulose distribution: Callose (**A**–**D**) and xyloglucans (**E**,**F**) detection 21 days post-infection (dpi) *M*. *incognita* induced galls of Col-0 (**A**,**C**,**E**) and *fra2* (**B**,**D**,**F**). Aniline blue staining (**A**,**B**), callose (**C**,**D**) and xyloglucans immunolabelling. Aniline blue staining (**A**) is more intense in the giant cells of Col-0 roots compared to *fra2* (**B**). Immunolabellling with anti-β-1,3-glucan follows the same pattern as that of aniline blue staining (**D** cf. **C**); inset in D: toluidine staining of panel D, note that the nematode is more intensively stained than the root tissues. The LM25 antibody was used to localize xyloglucans and showed a slightly increased signal in Col-0 contrast to *fra2* (**F** cf. **E**). Asterisks indicate giant cells in the nematode feeding site; N, nematode (*M. incognita* female). Scale bars: (**A**–**F**) 40 µm, (inset in **D**), 40 μm.

**Figure 6:**
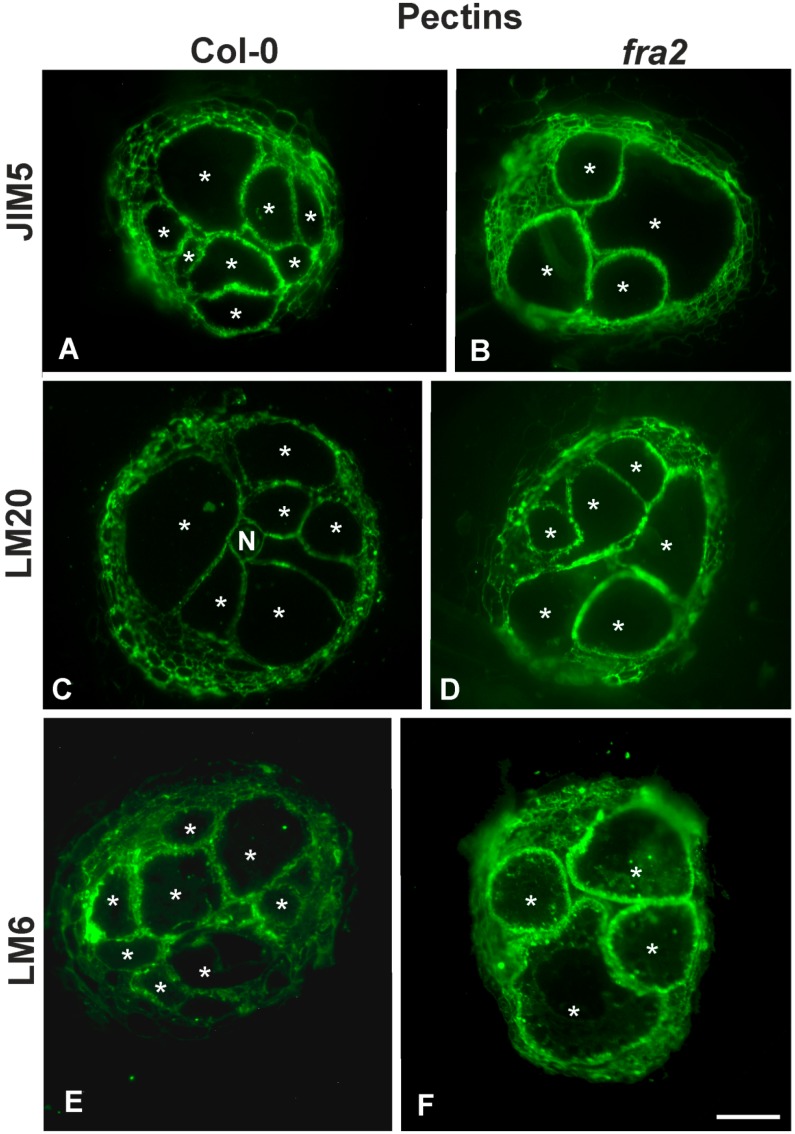
Pectin immunolabelling 21 dpi *M*. *incognita* induced galls of Col-0 (**A**,**C**,**E**) and *fra2* (**B**,**D**,**F**). DeSPHG localized by the JIM5 antibody did not seem to differ between Col-0 and *fra2* nematode-induced giant cells (**B** cf. **A**). MPHG recognized by the LM20 antibody were intensified in the *fra2* giant cell walls compared to Col-0 (**D** cf. **C**). Arabinan localized epitopes (LM6) had a weak signal in Col-0 roots (**E**) and a more intense signal in *fra2* root knots (**F**). Asterisks indicate giant cells in the nematode feeding site; N, nematode (*M. incognita* female). Scale bar: (**A**–**F**) 40 µm.

Transverse sections of Col-0 and *fra2* root knots 21 days post-infection with *M*. *incognita* (Figure 4, Figure 5 and Figure 6) were prepared for cell wall matrix polysaccharide immunodetection and comparative analysis. Calcofluor-white (Figure 4A,B) and toluidine blue staining (Figure 4C–F) allowed the observation of anatomical features, confirming that *M. incognita* induced the formation of giant cells (GCs) in both Col-0 and *fra2* roots. However, it seemed that the GCs formed in *fra2* were fewer compared to those formed in Col-0 (Figure 4D cf. Figure 4C), a trend visible in all gall cross sections. The GCs located in the vascular cylinder bared numerous nuclei, were highly vacuolated and appeared to be of similar morphology in both Col-0 and *fra2* plants (Figure 4E cf. Figure 4F). 

Callose was omnipresent in the GC cell walls of the Col-0 plants (Figure 5A,C) contrary to the GC cell walls of *fra2*, which lacked callose signal (Figure 5B,D) as revealed by both aniline blue staining and anti-β-1,3-glucan localization. On the other hand, no significant differences in the distribution pattern of the LM25 bound epitope were observed (Figure 5E,F).

MPHGs signal was more intense in the GC cell walls of *fra2* compared to the wild type (Figure 6D), while DeSPHG signal did not seem to differ among the roots of Col-0 and *fra2* (Figure 6B cf. Figure 6A). LM6 recognized epitopes were omnipresent in *fra2* nemadote-infected roots (Figure 6F) while a weak signal was observed in the corresponding infected Col-0 roots (Figure 6E). Table 1 summarizes all epitopes present in GCs. 

### 2.3. The Impact of Katanin Mutation on the Infection of A.thaliana by M. incognita

**Figure 7:**
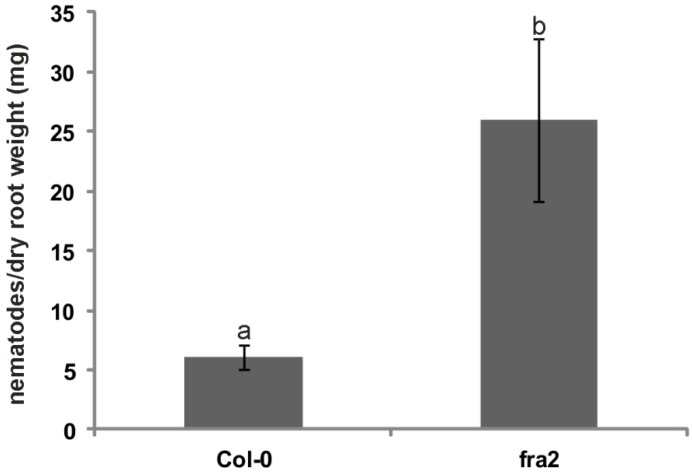
Total nematode burden in Col-0 and *fra2* expressed as female number/dry root weight (mg). Error bars represent standard error of a mean. For nematode counting, *n*  =  10 root systems from plants harvested on two separate occasions were used. Different letters indicate statistically significant difference (*p* < 0.01).

The total nematode burden between Col-0 and *fra2* expressed as female number/dry root weight (mg) significantly differed (*p* < 0.05). Root of *fra2* seemed to bear more nematodes per mg of their dry root weight compared to Col-0 (Figure 7) indicating that root invasion success was higher in *fra2* mutants.

## 3. Discussion

The fragile fiber 2 (*fra2*) mutant, as its name states, displays increased fragility in all the plant organs and particularly in the stems [22]. The reduced cellulose content and the altered lignin deposition, leads to shorter and thinner fibers and to lower mechanical resistance [22]. In the present study, we additionally observed that hemicellulose and pectin cell wall components have a different deposition pattern in the *fra2* mutant root compared to Col-0 (Figure 2 and Figure 3). Xyloglucans (Figure 2) but also MPHGs, DeSPHG and arabinans (Figure 3) had a differential distribution between Col-0 and *fra2*. 

Generally it was reported that the *fra2* root cell division zone is shorter than that of Col-0 [24]. This difference was not the outcome of a decrease in cell divisions rate, but was due to the distorted shape of the root cells of the mutant. The root cells at the division zone of the mutant are unable to elongate, contrary to those of the wild type, a fact that can be seen by checking the height/width ratio of the protodermal root cells. So, the failure of MT alignment in *fra2* results in a dwarf root phenotype and due to the cell width increase, an increase in root diameter was observed [24]. Although cell shape is definitely controlled by MTs arrangement, there is evidence on the participation of the cell wall matrix materials in the morphogenesis of various cell types [25,26]. There is strong evidence of interplay between MTs and various cell wall components [25]. MTs that cannot be correctly aligned may have an impact on the differential cell wall component organization, but also the establishment of a specific cell wall micro domain can possibly affect MT arrangement, indicating the tight connection between MTs and cell wall components [25]. So, the inability of the cells to acquire their shape could be the result not only of the altered MT order, but because also of the changes observed in the structure of the cell wall architecture. 

During root development, root cell walls must endure both the internal forces created by the growing protoplast and the external forces due to root soil penetration; consequently, root cell walls must be strong enough to withstand the applied forces but at the same time they must be flexible enough to support root growth. Therefore, cell wall synthesis, softening and maturation processes are co-ordinated [27] in order to support cell maturation and expansion. Especially cell expansion requires normal cellulose synthesis [28], but also pectin modifications [29,30]. Since we focused our study onn the root differentiation zone (visible xylem elements) [31], it is expected that, by the action of PMEs, the methyl-esterified homogalacturonans are demethylesterified. As already reported, DeSPHGs are interconnected by calcium bridges, forming cell wall matrices of high viscosity to support the cell wall stiffening in the differentiation zone [32]. Indeed, in Col-0 roots LM20 (MPHGs) signal was restricted to the vascular cylinder, “missing” from the other root tissues, in which cells could have acquired their final size. On the contrary, the extensive presence of the LM20 epitope in the root sections of *fra2* (Figure 3D), could possibly be a side-effect of the hampered development of *fra2* root [24,33]. Generally, MPHGs characterize cell walls which elongate (i.e cell walls of hypocotyl cells) [34]. In *fra2* root cells, the extensive presence of the LM20 epitope could indicate that the cells have not terminated their elongation process, since the desired cell shape has not yet been acquired.

The notion that cell elongation of *fra2* root cells is hampered is further on confirmed by the differential LM6 epitope distribution observed in *fra2* root sections compared to Col-0 (Figure 3E). Usually, arabinans are required for maintenance of cell wall flexibility [29] and their presence intensifies in the differentiation root zone [29]. This local cell wall remodeling reflects a change in the cell wall mechanical properties [29]. In *fra2* mutant this shift could be delayed indicating a hindered cell differentiation. Xyloglucans signal is distributed more broadly in *fra2* root sections (Figure 2), further defining the problematic cell expansion of *fra2* root cells. Xyloglucans binding to cellulose microfibrils seems to reinforce the cell wall, turning it more rigid and less expandable [35]. In *fra2* roots, the increased presence of xyloglucans, that interact with cellulose can result in increased cell wall rigidness, opposing flexibility (Figure 2D). Except for the disturbed root development, the altered hemicellulose and pectin deposition patterns observed in *fra2* could be the outcome of the plants compensatory efforts to overcome the potential stiffness reduction because of cellulose loss. Generally no important change in the elasticity modulus of the cell walls in katanin mutants was recorded, suggesting that other cell wall components compensate for the hampered cellulose synthesis observed in katanin mutants [36,37]. In this context, xyloglucans were essential when katanin expression was hindered [37]. 

The unique cells induced by the nematode infection in the feeding structure must be capable to withstand the turgor pressure within, whilst maintaining the necessary flexibility to cope with the periodic demands of nematode feeding [5]. To facilitate this, the walls of the cells produced in the feeding site are specifically modified. In previous studies, abundant MPHGs, xyloglucans and arabinans were found in syncytial cell walls of a cyst nematode [16,17]. In particular, it is reported that the cell walls in syncytia cells formed in *A. thaliana* are rich in MPHGs and in arabinans, while galactans were not detected. The cell wall modifications continue to take place even after the syncytium reaches its final size at 14 dpi. The abundance of MPHGs in the vascular cylinder of infected roots implies that the syncytial cell wall is flexible. Since the syncytia are highly metabolically active and represent a nutrient sink for solutes, their flexibility is essential for their functionality [38]. Apart from that, the cell walls of the GCs of *M. incognita* infected roots in three different hosts [11] display the same characteristics. The GCs are metabolically active and act as transfer cells for the nematode nourishment. A unique feature of the giant cells is their ability to grow isotropically. This kind of cell expansion requires extensive and coordinated cell wall remodeling. The loosening of the cellulose/xyloglucan network is mediated through the action of specific enzymes, like glucanases, expansins and endotransglycosylases. These subtle modifications provide the GCs cell walls with the necessary flexibility during the changes in cell volume and shape. In the present study, we found that callose was also present in the GCs cell walls, (and seemed to increase its abundance upon nematode infection Figure 2 cf. Figure 5) as confirmed by both aniline staining and anti-β-1,3-glucan immunolabelling (Figure 5). It is commonly accepted that callose controls the permeability of plasmodesmata and/or provides mechanical support to the cell wall against different environmental stresses. This polysaccharide is synthesized upon injury, low temperature, heavy metals and various pathogens [39,40,41,42]. Another biotic stress upon which callose is synthesized, seems to be nematode infection adding the necessary mechanical support to the GCs cell walls structure, along with the other cell wall changes ([11]; this study). However, one cannot exclude the fact that callose could also act as a defense barrier as reported for other nematode species [10]. 

In order to elucidate if the observed differences in GC cell wall composition had any functional consequences on the plant-nematode interaction, we used the *fra2* mutant, a mutant with established cellulose production defects [22]. Cellulose microfibrils are the scaffold of the cell wall and together with hemicelluloses they provide the cell wall with the necessary rigidity [43]. The *fra2* mutant cell walls are extremely fragile; this fragility, due to reduced cellulose production, could lead to an increased susceptibility towards *M. incognita* infection, since fragile cell walls could lead to easiest nematode infection and penetration. In fact, susceptibility experiments proved that *fra2* is more vulnerable to *M. incognita* infection than Col-0 (Figure 7). *M. incognita* females were able to induce a feeding site in the *fra2* roots more successfully than they did in Col-0. Conclusively, it seems possible that katanin MT severing is necessary for plant defense against nematode infection, even in an indirect way, adding to the existing microtubule associated proteins (MAPs; e.g., MAP65-3; [44]) that have a pivotal role in RKN invasion. Moreover, it was reported that MT reorganization is essential for feeding site establishment and juvenile maturation [45]. In accordance to this, katanin, a MT arrangement mediator, [33] seems to also have an important contribution towards *M. incognita* infection. 

The established/observed ([11]; this study) cell wall modifications in GC cell walls differed in *fra2* plants (Figure 5 and Figure 6). Table 1 summarizes the noticed cell wall modifications that took place upon nematode infection in both Col-0 and *fra2* roots. A noticeable difference refers to the levels of callose in *fra2* GC cell walls. As confirmed by aniline staining and anti-β-1,3-glucan immunolabelling callose was absent from *fra2* GC cell walls (Figure 5). Generally, callose is responsible for cell wall flexibility supporting tenseness and contraction during mechanical stress. In other words, this polysaccharide decreases firmness and increases elasticity of cell walls [46]. Accordingly, *fra2* GC cell walls lack elasticity, due to callose absence, becoming more vulnerable to turgor pressure. However, this loss seems to be compensated by the increased levels of arabinan (LM6; Figure 6) and MPHGs, which both generally provide cell wall with the necessary flexibility ([11,17]; this study). Therefore, the wall flexibility of the GCs is maintained, even in a mutant with reduced cell wall elasticity (*fra2*). Cell wall alterations are mediated by the nematode-induced regulation of host genes [6]. It seems therefore plausible that the nematode recognizes the peculiarities of different hosts and is capable to manipulate host gene expression to successfully establish a feeding site. This notion is further supported by the already reported differential response of different hosts to *M. incognita* infection [11].

## 4. Materials and Methods 

### 4.1. Plant Growth Conditions, Nematode Rearing and Collection

Seeds of *Arabidopsis thaliana* L. (Heynh) (as previously reported; [47]) wild type (ecotype Columbia; Col-0) and *fragile fiber 2* (*fra2*) mutant (purchased from the NASC European Arabidopsis Stock Centre, Nottingham, UK) were surface sterilized with a 30% (*v*/*v*) bleach solution and kept at 4 °C for 48 h. Subsequently, the seeds were transferred in soil and left to germinate and grow in a growth chamber under a constant 16 h day/8 h night regime at an ambient temperature of 21 ± 1 °C, with light intensity set at 120 μmol m^−2^·s^−1^.

Seeds of tomato plants *Solanum lycopersicum* L. cv Belladonna were directly planted in pots filled with soil and grown in the same conditions as stated above for two months prior to nematode infection. Populations of *M. incognita* of Greek origin were reared on the above tomato plants [48]. Freshly hatched (24–48 h) J2 were extracted from egg masses according to a modified Baermann technique [49] from 40 day-old (d) infested roots, to be used for the bioassays. 

### 4.2. Light and Fluorescence Microscopy

All chemicals and reagents used were purchased from Sigma (St. Louis, MO, USA), Merck (St. Louis, MO, USA), and Applichem (Darmstadt, Germany), unless otherwise stated. Twenty one days post infection (21 dpi), root tissue, of infected and non-infected (control) Col-0 and *fra2* plants, was carefully cleared from soil. Given the incubation conditions, at 21 dpi giant cells are fully formed [50], a fact also verified in *A. thaliana* plants infected with *M. incognita* [6]. Both root knots and uninfected root pieces were prepared for light microscopy according to [51]. Each experiment was conducted in three replicates and about 10–15 roots from each experiment were selected and further on processed. In short, root tissue pieces were fixed in 2% *w/v* paraformaldehyde (PFA) and 0.5% *v/v* glutaraldehyde (GA) in PEM buffer (50 mM PIPES, 5 mM EGTA, 5 mM MgSO_4_, pH 6.8) for 2 h at 4 °C. Fixed roots were rinsed twice in the same buffer for 10 min. Root tissue samples were then dehydrated in a graded ethanol series, subjected to post-fixation in 0.25% OsO_4_ in 30% ethanol and embedded in LR White resin. Finally, the samples were sectioned using an ULTROTOME III TYPE 8801A ultramicrotome (LKB, Stockhom, Sweden) equipped with a glass knife. Semi-thin sections (0.5–2 μm) were stained with 0.5% (*w/v*) toluidine blue O and observed by light microscopy. Especially for the uninfected roots, special care was taken to obtain sections at the differentiating zone [52], in both Col-0 and *fra2*. Since this particular root zone for *fra2* is observed closer to the meristem than that of Col-0 [24] we used the visible xylem elements as a marker of root differentiation. Considering root knots special care was taken to obtain transverse sections through the middle of the gall in order to obtain vision of the biggest giant cells [53]. 

Detection of callose by aniline blue fluorochrome was performed by semithin sections remaining in aniline blue solution (0.05% (*w/v*) aniline blue) in 0.07 M K_2_HPO_4_ buffer, pH 8.5) [54,55] and observed under an epifluoresence microscope. For callose detection via immunolabeling, semithin sections (approximately 2–3 μm thick) of material embedded in LRW resin were transferred to glass slides and blocked with 5% (*w/v*) BSA in PBS for 5 h. After washing with PBS, anti-callose antibody [56] diluted 1:40 in PBS containing 2% (*w/v*) BSA was applied overnight. Following rinsing with PBS and blocking again with 2% (*w/v*) BSA in PBS, the sections were incubated for 1 h, at 37 °C in FITC anti-mouse IgG diluted 1:40 in PBS containing 2% (*w/v*) BSA. After rinsing with PBS, the sections were mounted using an anti-fade medium or with calcofluor white [57].

All of the fluorescence specimens were examined with an Axioplan microscope (Zeiss, Berlin, Germany) equipped with a UV source, a Differential Interference Contrast (DIC) optical system. All the photos were taken with a Zeiss Axiocam MRc5 digital camera using the ZEN 2.0 software (Zeiss, Berlin, Germany) according to the manufacturers’ instructions [58]. Two filter sets were used for specimen observation: a filter set provided with exciter solid glass filter 365 nm and barrier long-wave pass band filter 420 nm, and another set provided with exciter pass band filter 450–490 nm and barrier pass band filter 515–565 nm. All samples were checked for UV autofluorescence using the above filters. Digital micrographs were processed with Adobe Photoshop (Adobe Inc., San Jose, CA, USA) with only linear settings.

### 4.3. Cell Wall Epitope Immunolabeling

Another set of semi-thin sections were incubated with rat monoclonal antibodies directed to cell wall matrix polysaccharides according to [11] (Table 2). Τhe distribution of highly esterified HGs recognized by LM20 antibody [59], the partially de-esterified HGs recognized by JIM5 antibody [60], the motifs of xyloglucans recognized by LM25 [61] and arabinan motifs with the LM6 antibody [59]. Pectate lyase treatment before immunolabeling with LM25 was performed as described by [11]. Semi-thin sections (approximately 2–3 μm thick) of material embedded in LRW resin were transferred to glass slides and blocked with 2% (*w/v*) BSA in PBS for 2 h. After washing with PBS, a specific antibody against a cell wall matrix material was added and applied overnight. All antibodies were diluted 1:40 in PBS containing 2% (*w/v*) BSA. Following rinsing with PBS and blocking again with 2% (*w/v*) BSA in PBS, the sections were incubated for 2 h, at 37 °C with a FITC anti-rat IgG (Sigma) diluted 1:40 in PBS containing 2% (*w/v*) BSA. After three washes with PBS, the sections were mounted using an anti-fade medium containing *p*-phenylenediamine.

### 4.4. Arabidopsis Wild-Type and fra2 Mutant Nematode Susceptibility 

Artificial infestation with *M. incognita* was performed on *A. thaliana* wild-type and *fra2* mutant plants using 1000 J2/plant. The plants were maintained for the completion of one biological cycle, which is 40 dpi, at the same conditions as stated above. After that, roots were stained with acid fuchsin [62] and the following variables were assessed: root weight (dry) and total number of female nematodes per gram of root at 10× magnification under uniform illumination by transparent light within tissue sample. The experiment was performed twice, and the treatments were always arranged in a completely randomized design with five replicates. Because ANOVA indicated no significant treatment by time interaction, means were averaged over experiments. Nematode infestation levels were compared using Tukey’s test at *p* ≤ 0.05. Statistical analysis was performed using SPSS 20 (IBM, Armonk, NY, USA).

## 5. Conclusions

The MT katanin severing defects of *fra2* mutants lead to altered pectin and hemicellulose distribution, compared to Col-0. Xyloglucans and MPHGs were more abundant in *fra2* a fact that could lead to an increased *M. incognita* infection rate. Following *M. incognita* infection in *fra2* roots, the GC cell wall modifications (highly-esterified pectic homogalacturonan and pectic arabinan) were present, maybe to compensate for the low amount of callose, which was omnipresent in GC cell walls of Col-0. Therefore with this study we conclude for the first time that katanin MT severing seems important in plant defense against *M. incognita*. The nematode however, seems to be able to overcome this “katanin deficiency” and eventually induce the necessary GC cell wall modifications and to establish a feeding site.

## Figures and Tables

**Table 1:** Overview of the studied cell wall components present in GC cell walls.

Cell Wall Components		*A. thaliana*	
		Col-0 GCs	*fra2* GCs
Hemicelluloses	Xyloglucans	+/+	+/+
	Callose	+/+	−
Pectins	MPHG	+/−	+/+
	DeSPHG	+/+	+/+
	Arabinan	+/−	+/+

**−**: weak presence; +/**−**: intermediate presence; +/+: strong presence.

**Table 2:** List of the monoclonal antibodies used in the current study, of epitopes they recognize and of references.

Antibody	Epitope	References
*Hemicelluloses*		
LM25	Recognizes XLLG, XXLG and XXXG motifs of xyloglucans	[61]
Anti-β-1,3-glucan	Recognizes 1,3 β-glucan	[57]
*Pectins*		
LM20	HG domain in pectic polysaccharides, requires methyl esters for recognition of HG and does not bind to unesterified HG	[59]
JIM5	HG domain of pectic polysaccharides, recognizes partially methyl-esterified epitopes of HG, can also bind to unesterified HG	[60]
LM6	Recognizes a linear pentasaccharide in (1-5)-α-L-arabinans	[59]

## Abbreviations

RKN	Root-Knot Nematodes
GCs	Giant Cells
MT	Microtubules
DeSPHG	De-esterified Pectic HomoGalacturonan
MPHG	Methylesterified Pectic HomoGalacturonan
